# Impact of Neurological Rehabilitation in Autoimmune Encephalopathy: A Case Report

**DOI:** 10.7759/cureus.50466

**Published:** 2023-12-13

**Authors:** Sharvil Nerkar, H V Sharath, Shraddha S Kochar, Sarang S Bhoyar

**Affiliations:** 1 Paediatric Physiotherapy, Ravi Nair Physiotherapy College, Datta Meghe Institute of Higher Education and Research, Wardha, IND

**Keywords:** coordination, physical therapy, neurorehabilitation, neurology, autoimmune encephalitis

## Abstract

The physiotherapy rehabilitation program aims to build up functional activity, coordination, and balance ambulation for proper independence in the activities of daily living. An uncommon but complicated neurological condition called autoimmune encephalopathy is caused by the body’s immune system mistakenly attacking healthy brain tissue. This condition often leads to a wide range of neurological and cognitive symptoms, making its diagnosis and treatment challenging. Physiotherapy, a vital component of the comprehensive management of autoimmune encephalopathy, plays an important role in improving the health of affected patients. In this report, the patient’s occupational requirements and enhancement in executing daily living tasks were the focus of physiotherapeutic rehabilitation. The outcomes included the Berg Balance Scale and Functional Independence Measure. We observed a marked increment in muscle tone and strength, active range of motion, and significant enhancement in the individual’s functional independence with physiotherapeutic protocol postoperatively. This case report provides an overview of the execution and function of physiotherapy in the management of autoimmune encephalopathy, emphasizing its contributions to symptom alleviation, functional recovery, and the overall well-being of patients.

## Introduction

Autoimmune encephalitis (AIE) is an immune-mediated state that causes inflammation in the brain and is among the main reasons for non-infectious encephalitis. AIE acts on multiple neural system components, including the entire cerebrospinal axis, the limbic system, and/or the spinal cord. The prevalence of adult encephalitis varies from 0.7 to 12.6 cases per 100,000 people and has been reported in adult and pediatric populations. AIE’s specific immunological resistance-breaking mechanism remains mostly unspecified. AIE cases frequently have a history of infection, which causes inflammation and results in neurological symptoms [1]. AIE syndromes linked to antibodies on the sensitivity to immunosuppression set them apart on the cell surface and a preference for the limbic system. AIE linked to intracellular antibodies usually reacts poorly to immunosuppression and can target a wider breadth of the nervous system [2]. Immunosuppressive responsiveness and limbic system preference are characteristics of AIE syndromes associated with cell-surface antibodies. AIE linked to intracellular immunoglobulin, on the other hand, tends to attack a larger region of the nervous system and is generally less sensitive to immunosuppression [3]. The identification of correlated anti-neuron antibodies is necessary for diagnosis, and immune-suppressive medications are used in conjunction with neoplasia treatment for care [4].

Similar to AIE and cerebellitis, no other subspecialty of neurology has seen a similar increase in clinically significant serological biomarkers over the last decade. Numerous recently identified neuronal autoantibodies help in the prediction of prognosis, early diagnosis, and treatment selection [5]. First-line treatment, which aims to reduce antibody levels quickly, normally comprises intravenous corticosteroids often combined with intravenous immunoglobulin (IVIG) or plasma exchange [6]. During the acute stage, disorientation, confusion, confabulation, and amnesia are common symptoms of encephalitis patients. Movement disorders can exhibit a wide range of manifestations in AIE syndromes. Patients suffer from limited tremors, dystonia, and chorea in combination. Although they affect all limbs, the face and mouth are the most commonly affected [7]. Because magnetic resonance imaging (MRI) has a higher sensitivity and specificity for evaluating encephalitis than computed tomography (CT) scans, it is the preferred methodology for neuroimaging [8]. Functional abilities can be restored using individualized physiotherapy treatment. This study focuses on how patients with motor deficits respond to long-term rehabilitation along with associated visual deficits, seizures, and cognitive impairment [9]. The individual’s quality of life is further impacted by the impairment in activities of daily living resulting from the clinical symptoms associated with AIE. So far, physical therapy has proven to be advantageous. Maintaining breathing, circulation, and airway integrity is essential [10]. The findings indicate that core strengthening and pelvic proprioceptive neuromuscular facilitation (PNF) have a complementary effect on gait, trunk impairment, and balance in AIE patients [11].

Rehabilitation is beneficial for a large number of encephalitis survivors. Unfortunately, there is no thorough analysis that details the results of rehabilitative interventions for both adults and children who have infectious encephalitis at this time [12]. A wide range of immune-suppressive medications, as well as those that target antibody-mediated disease pathogenesis mechanisms, are available for treating AIE [13]. Several encephalitis patients have long-term multidisciplinary care due to lingering physical or neurological shortcomings [14]. IVIG, therapeutic plasma exchange (TPE), anti-epileptics, immunosuppression, and intravenous corticosteroids are used to treat AIE [15]. AIE is a probably curable immune-mediated state that is becoming more common [16]. However, clinical practice has yet to keep up with advances from the lab bench, resulting in a large knowledge gap and numerous unanswered questions about both short-term and long-term AIE management [17]. A second-line medication called rituximab is being used increasingly to treat various forms of AIE and seems to be working [18].

## Case presentation

Patient information

A 52-year-old male patient, a farmer by occupation, admitted on August 21, 2023, to Acharya Bhave Rural Hospital, presented with the chief complaint of low-grade fever for five days and suddenly suffered from an altered level of consciousness and weakness. He had experienced his first convulsion on the 10th day of admission to the neurology intensive care unit (ICU). After that, he experienced four episodes of intermittent seizures at an interval of one month. This symptom was insidious and progressive, and he arrived at the hospital after being admitted to the neurology ward, developing psychosis with hyperactivity. There was no other relevant recent medical history or past history. He did not provide a history of any comorbidities or bladder or bowel complaints. Presently, the patient complained of generalized weakness, headache, psychosis, and difficulty in speech. On September 9, 2023, physiotherapy rehabilitation was started for the above-mentioned complaints. The timeline is depicted in Table 1.

**Table 1:** Timeline of the patient.

Events	Dates
Date of admission	21/8/2023
Date of surgery	27/8/2023
Date of physiotherapy rehabilitation	8/9/2023
Date of discharge	15/10/2023
Date of follow-up	29/11/2023

Clinical findings

Before the commencement of the examination, informed consent was taken from the patient, and he was examined. On examination, he was cooperative and well-oriented to person, place, and time. The patient was afebrile and hemodynamically stable. He was seen in a supine lying position with head end elevated to 30° and knee and ankle supported with pillows under. He was mesomorphic, with a basal metabolic rate of 24 kg/m^2^. His speech was altered, but his vision and hearing were normal. During a neurological evaluation, sensations were intact. The tone and muscle strength were reduced in the superior and inferior extremities. All deep tendon reflexes were diminished. Babinski’s sign was positive. The patient was unable to stand and walk. As evaluated by the Functional Independence Measure (FIM), the patient necessitated maximal assistance with basic activities of daily living (ADLs) (bathing, eating, toileting, and transferring) as well as instrumental ADLs (transportation, communication, and medication handling).

Diagnostic assessment

**Figure 1:**
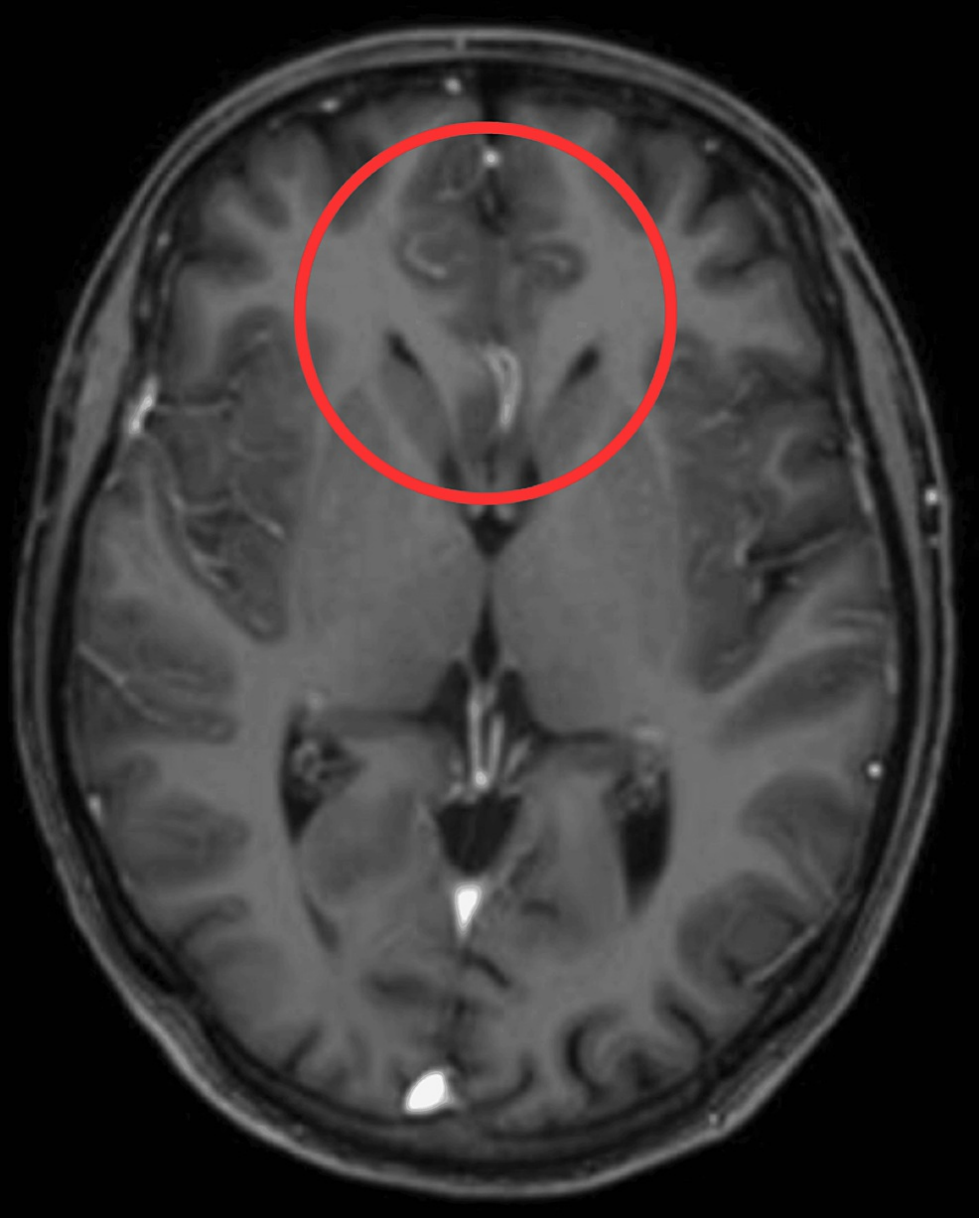
Magnetic resonance imaging of the brain. The red circle shows the non-enhancing symmetrical signal abnormalities with diffusion restriction in the bilateral parasagittal frontal region involving the cingulate gyrus.

A complete blood count (CBC) and cerebrospinal fluid (CSF) examination were done. White blood cells and C-reactive protein were elevated. MRI of the brain was performed, as shown in Figure 1. The above features favored a diagnosis of acute hyperammonemia or acute autoimmune encephalopathy rather than an ischemic.

Physiotherapy intervention

**Figure 2:**
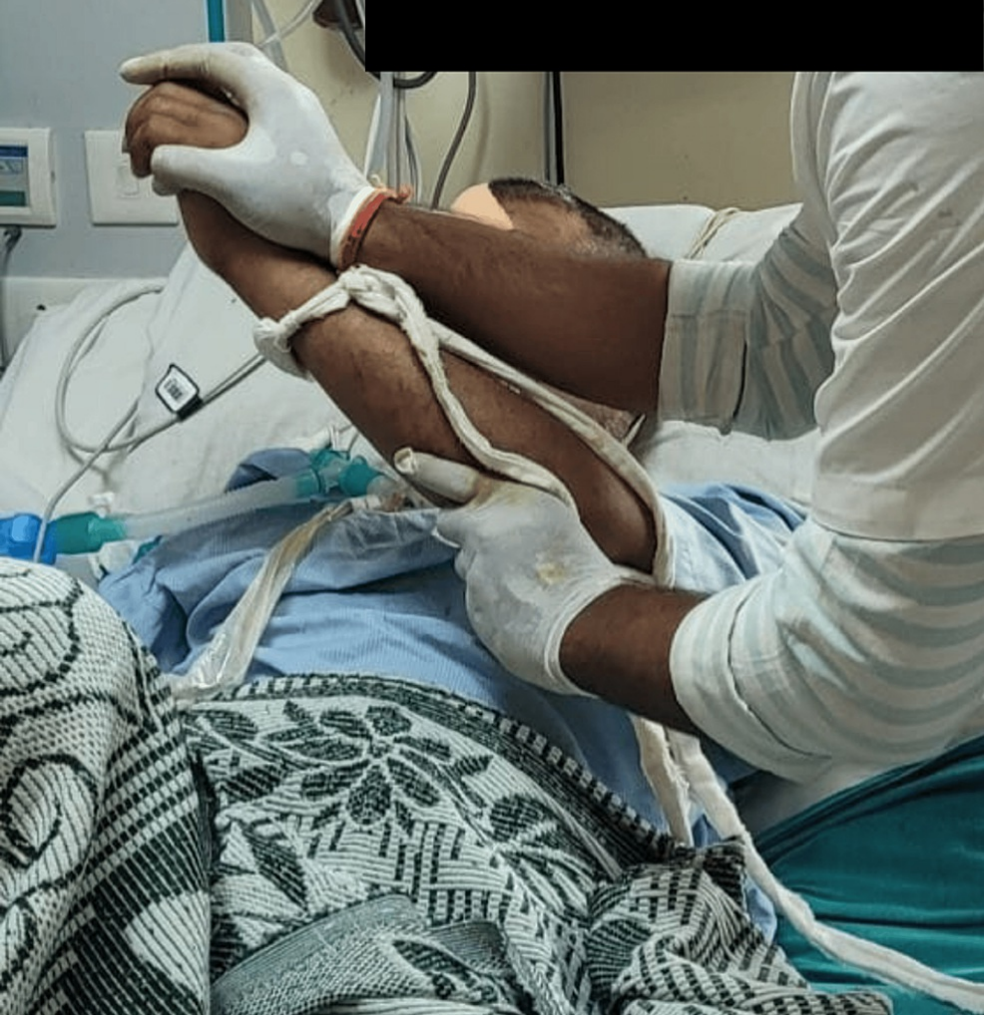
Proprioceptive neuromuscular facilitation of the left upper limb.

**Figure 3:**
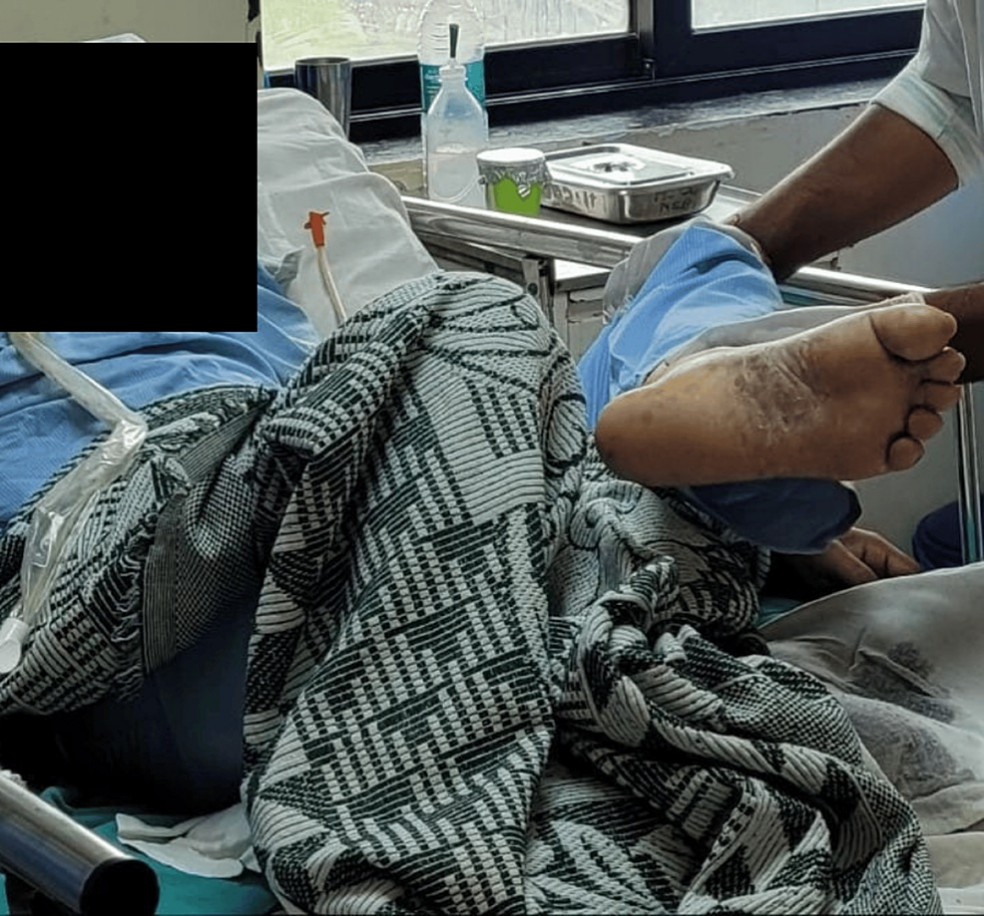
Proprioceptive neuromuscular facilitation of the right lower limb. PNF: Proprioceptive Neuromuscular Facilitation

Table 2 shows the physical therapy protocol administered to the patient. Figure 2 and Figure 3 show the patient being rehabilitated using PNF techniques.

**Table 2:** Rehabilitation protocol. N/A: not applicable; PNF: proprioceptive neuromuscular facilitation; reps: repetition

Problem identified	Goal	Treatments strategy	Intervention	Progression (4 weeks)
Patient and family education	To make the patient do physiotherapy exercises actively and promote positive attitudes toward treatment	Effective conversation of the therapist involving the patient and his family in the physical therapy session	The patient, along with his family, was explained the importance of physiotherapy and the treatment protocol	Home program
Unable to perform functional activity	To promote functional activity	Functional mobility exercises	Mat exercises	N/A
Poor balance and coordination	To maintain balance and coordination	To gain static and dynamic balance with coordination	Stretching, functional reach, sitting to standing, standing with closed eye	N/A
Problems in the trunk and pelvic control	To develop trunk and pelvic stability	Core stability and strength exercise	Pelvic PNF contract-relax. Pelvic bridging exercise (10 reps × 1 set)	Pelvic and trunk muscle strengthening on therapeutic ball
Decrease muscle tone	To build muscle tone	Mobilization technique for tone development	Joint approximation (10 reps × 1 set)	Joint approximation (10 reps × 2 sets)
Minimize and decrease muscle strength and endurance	To increase and build muscle strength and endurance	Resistance exercise with PNF	PNF for upper and lower limb (10 reps × 1 set), strengthening exercise with ½ kg weight cuff. (10 reps × 1 set), resistance band exercise	Strengthening exercise with 1 kg weight cuff
Bed mobility hampered	To improve bed mobility	Active and passive movement exercise	Rolling mobilizing from supine to long sitting, vertical transfer	Unsupported sitting, unsupported standing
Cognitive impairment	To increase memory and attention	Brain exercises	Puzzle-solving, music therapy, counting	Drawing, writing
Affected gait pattern	To improve walking pattern	Gait training	Supported treadmill walking (1 minute)	Unsupported walking (1 minute)
Impaired gross and fine motor skills	To gain gross and fine motor skills	-	Stretching spring press (10 reps × 1 set), soft ball squeeze (10 reps × 1 set)	(10 reps × 2 sets) after month
Affected speech	To improve speech	Oromotor training	Jaw exercise. Stroking with brush lip exercise	Tong range of motion exercise. Vocal exercise

Follow-up and outcome measures

Table 3 shows the manual muscle testing (MMT) before and after the treatment protocol. Table 4 and Table 5 show the deep tendon reflex and muscle tone, respectively, pre- and post-treatment. Table 6 illustrates the outcome measures taken before and after the treatment.

**Table 3:** Manual muscle testing. 0: no contraction; 1: flickering contraction; 2: full range of motion (ROM) with gravity eliminated; 3: full ROM against gravity; 4: full ROM against gravity with minimum resistance; 5: full ROM against gravity with maximum resistance

Muscles	Pre-treatment	Post-treatment
Shoulder flexors	2/5	4/5
Shoulder extensor	1/5	4/5
Shoulder abductors	1/5	4/5
Elbow flexors	1/5	4/5
Hip flexors	1/5	3/5
Hip extensors	1/5	3/5
Hip abductor	1/5	3/5
Hip adductor	1/5	3/5
Knee flexors	2/5	5/5
Knee extensors	2/5	5/5

**Table 4:** Deep tendon reflexes. +: diminished reflex; ++: normal reflex; +++: exaggerated reflex

Reflex	Pre-intervention reflex		Post-intervention reflex	
	Right	Left	Right	Left
Bicep jerk	+	+	++	++
Tricep jerk	+	+	++	++
Knee jerk	+	+	++	++
Ankle jerk	+	+	++	++
Jaw jerk	+	+	++	++
Planter reflex	Extensor	Extensor	++	++

**Table 5:** Muscle tone. TGS: Tone Grading Scale; 1+: decreased tone; 2+: normal tone; 3+: increased tone

Muscle group	Pre-treatment	Post-treatment
Shoulder flexors	1+	2+
Shoulder extensor	1+	2+
Shoulder abductor	1+	2+
Elbow flexors	1+	2+
Hip flexors	1+	2+
Hip extensors	1+	2+
Hip abductor	1+	2+
Hip adductor	1+	2+
Knee flexors	1+	2+
Knee extensor	1+	2+

**Table 6:** Outcome measures. WHO-QOL: World Health Organization-quality of life

Scale	Pre-treatment score 1^st^ week	Post-treatment score 6^th^ week
Mini-Mental State Examination	10/30	25/30
Functional Independence Measure	15/126	75/126
Berg Balance Scale	15/56	40/56
Glasgow Coma Scale	8/15	12/15
WHO-QOL	46/100	70/100
Environmental performance	45/100	70/100

## Discussion

During the management and rehabilitation of AIE, early physical therapy can help patients achieve easy and fast recovery from autoimmune encephalopathy. This rare neurological condition involves the immune system attacking the central nervous system, leading to a range of cognitive, physical, and behavioral symptoms. Physiotherapy can be a valuable part of the complete care plan for these patients. AIE can result in muscle weakness, tremors, and coordination problems. Physiotherapy can assist patients in regaining control of their movements, improving and increasing muscle strength, and enhancing balance and coordination along with cognitive skills. Through targeted and conducted exercises and therapies, physiotherapists can assist in restoring motor function, making daily activities more manageable. Patients with AIE often face difficulties with mobility. Physiotherapy interventions, such as gait training and assistive devices, can promote independent mobility. There are no formal guidelines for physiotherapy programs for individuals with encephalitis. There is a lack of neuroscientific research on the positive impacts of physiotherapy protocol on individuals suffering from this disease [18].

Enhancing the patient’s mobility and independence is another goal of physical therapy programs. The exercises are provided in phases, beginning with bed rotations, sleeping to sitting, sitting balanced, sitting to standing, and balanced standing exercises [19]. AIE can have enduring outcomes. The continuous guidance and support from physiotherapists help patients manage their condition over the long term, adapting the treatment plan as needed. Collaboration between various healthcare professionals, including neurologists, neuropsychologists, occupational therapists, and speech therapists, is crucial in managing AIE. Physiotherapists play an essential role in this multidisciplinary approach, ensuring holistic care for the patient [20].

## Conclusions

This case report highlights the significant impact of neurological rehabilitation in the context of autoimmune encephalopathy. The patient’s journey underscores the importance of a multidisciplinary approach, involving neurologists, physiotherapists, and healthcare professionals. Through targeted interventions and a personalized rehabilitation plan, substantial improvements were observed in the patient’s cognitive and motor functions. Neurological rehabilitation plays a crucial role in mitigating the debilitating effects of autoimmune encephalopathy, contributing to enhanced quality of life and functional independence. The case underscores the resilience of the nervous system and the potential for recovery even in the face of challenging autoimmune conditions. As we continue to explore the intricate relationship between autoimmune disorders and neurological function, this case report provides valuable insights into the efficacy of rehabilitation strategies. Further research and clinical studies are warranted to better understand the nuances of autoimmune encephalopathy and refine rehabilitation protocols for optimal patient outcomes. Ultimately, this case report emphasizes the need for a holistic and patient-centered approach to managing autoimmune encephalopathy, recognizing the pivotal role of neurological rehabilitation in restoring and optimizing neurological function.
